# Promoting Drowning Preventive Behaviors Among Caregivers of Children Under 5 Years Through a Health Belief Model-Based Educational Program in Thailand: A Community Study

**DOI:** 10.3390/healthcare14162619

**Published:** 2026-08-19

**Authors:** Lawitra Kawee, Phubate Napatpittayatorn, Chakkrit Ponrachom

**Affiliations:** 1 Department of Disease Control, Ministry of Public Health, Nonthaburi 11000, Thailand; kookkik.lawitra@gmail.com; 2 Faculty of Education, Kasetsart University, Bangkok 10900, Thailand; phubate.n@ku.th; 3 Faculty of Health Sciences, Siam Technology College, Bangkok 10600, Thailand

**Keywords:** drowning prevention, caregivers, children under five years, Health Belief Model, community intervention, quasi-experimental study, Thailand

## Abstract

**Highlights:**

**What are the main findings?**

- A Health Belief Model-based community educational program was associated with greater short-term improvements in caregivers’ health beliefs and self-reported drowning prevention behaviors than routine care after adjustment for baseline scores.
- Caregivers who participated in the Be SAFE Program showed significantly greater improvements in perceived susceptibility, perceived severity, perceived benefits, cues to action, and self-efficacy and lower perceived barriers than those receiving routine care.

**What are the implications of the main findings?**

- Structured community-based educational programs grounded in behavioral theory may strengthen caregivers’ capacity to adopt drowning prevention practices for young children.
- The Be SAFE Program represents a feasible approach that may be integrated into routine maternal and child health services in similar community settings, although larger studies with longer follow-up are needed to confirm its effectiveness.

**Abstract:**

**Background/Objectives**: Drowning among children younger than five years remains a major public health problem in Thailand, particularly in residential water environments. Caregivers’ supervision and home safety practices play an important role in preventing childhood drowning. This study evaluated the association between participation in the Be SAFE Program, a community-based educational intervention developed using the Health Belief Model (HBM), and changes in caregivers’ health beliefs and self-reported drowning prevention behaviors. **Methods**: A quasi-experimental pretest–posttest control group study was conducted among 60 caregivers of children younger than five years recruited from two community settings in Ubon Ratchathani Province, Thailand. Participants were assigned to either an intervention group (*n* = 30) or a control group (*n* = 30). The intervention group participated in the HBM-based Be SAFE Program, whereas the control group received routine care. Outcomes included HBM constructs and self-reported drowning prevention behaviors. Baseline-adjusted analyses of covariance (ANCOVA) were performed to compare post-intervention outcomes between groups. Holm adjustment was applied to account for multiple comparisons. **Results**: After adjustment for baseline scores, caregivers in the intervention group demonstrated significantly greater improvements than those in the control group in perceived susceptibility, perceived severity, perceived benefits, cues to action, self-efficacy, and self-reported drowning prevention behaviors, together with significantly lower perceived barriers (all Holm-adjusted *p* < 0.05). Large effect sizes were observed across all measured outcomes. **Conclusions**: The findings suggest that the HBM-based Be SAFE Program was associated with greater short-term improvements in caregivers’ health beliefs and self-reported drowning prevention behaviors than routine care. These findings support the potential value of theory-based community educational programs for childhood drowning prevention while highlighting the need for larger multi-site studies with longer follow-up to confirm the program’s effectiveness and broader applicability.

## 1. Introduction

Drowning remains a major global public health problem and is one of the leading causes of injury-related mortality among children under five years of age. Approximately 300,000 people die from drowning each year, with more than 90% of these deaths occurring in low- and middle-income countries [1,2,3]. In Thailand, childhood drowning remains an important cause of preventable mortality despite ongoing national prevention efforts [4]. Most fatal incidents involving young children occur in or around the home and in nearby natural or artificial water bodies, highlighting the importance of community-based prevention strategies that address household and caregiver-related risk factors [5,6].

Young children are particularly vulnerable because of their immature motor and cognitive development, limited ability to recognize hazards, and dependence on adult supervision [7,8,9]. Household water containers and other accessible water sources may also pose substantial risks to young children [10]. Beyond mortality, non-fatal drowning can result in severe neurological injury, long-term disability, and substantial consequences for survivors and their families [11]. These risks underscore the importance of prevention strategies that address both environmental hazards and the behaviors of caregivers responsible for young children.

Childhood drowning is influenced by interacting child, environmental, social, and caregiver-related factors. Environmental risks include unrestricted access to water sources, inadequate barriers or fencing, and unsafe residential environments, while social conditions such as poverty, limited caregiver education, family stress, and inadequate childcare arrangements may further increase risk [12,13,14,15,16,17,18]. Caregiver-related factors are particularly important because effective supervision, environmental management, and timely responses to emergencies are central to drowning prevention among young children [17,19]. Evidence indicates that drowning incidents may occur when caregivers are temporarily distracted by household chores, phone use, work responsibilities, or fatigue [19]. Thus, caregivers’ preventive capacity depends not only on knowledge but also on psychosocial factors such as caregiving burden, competing responsibilities, perceived competence, and family support. Interventions that strengthen caregivers’ confidence, motivation, and behavioral skills while addressing perceived barriers may therefore be more effective than knowledge-based education alone.

Several strategies have been recommended to reduce childhood drowning, including environmental modification, enhanced supervision, public awareness, swimming and water-safety skills, and community-based educational interventions [12,20]. Caregiver-focused education is particularly relevant because it can improve risk awareness, perceptions, confidence, and preventive practices related to child safety [9,20,21]. Evidence from interventions addressing childhood home injuries also suggests that educational approaches can improve safety practices among families with young children [22]. Community-based and family-oriented approaches may be particularly valuable when they incorporate active participation and practical learning. For example, recent work has explored parent- and child-focused approaches to drowning prevention education, including community-oriented educational activities [23]. However, educational interventions are likely to have greater potential for sustained behavioral change when they are guided by an established behavioral theory.

The Health Belief Model (HBM) is one of the most widely used behavioral theories in health promotion. It proposes that individuals are more likely to engage in preventive behaviors when they perceive themselves as susceptible to a health threat, recognize its severity, perceive greater benefits than barriers, receive cues to action, and have confidence in their ability to perform the recommended behavior. Previous systematic reviews and intervention studies have demonstrated that HBM-based interventions can improve preventive behaviors across various public health contexts, including injury prevention, child health promotion, and community health behaviors [24,25,26,27]. This framework is particularly relevant to drowning prevention because caregivers must continuously recognize environmental risks, overcome practical barriers, and maintain appropriate supervision during routine childcare. Strengthening caregivers’ health beliefs, self-efficacy, and behavioral skills may therefore increase their capacity and motivation to consistently implement drowning prevention practices. However, previous drowning prevention interventions have often emphasized knowledge provision or general safety education, with comparatively limited attention to theory-driven behavioral change among caregivers in community settings [21,23].

Despite increasing attention to childhood drowning prevention, several knowledge gaps remain. First, relatively few community-based interventions have specifically targeted caregivers of children under five years of age using a comprehensive behavioral theory. Second, previous studies have predominantly focused on knowledge or general safety practices, with less attention to changes in multiple behavioral determinants, particularly self-efficacy and perceived barriers. Third, evidence regarding HBM-based drowning prevention programs implemented through Thailand’s primary healthcare system, with active involvement of Village Health Volunteers (VHVs), remains limited. Addressing these gaps is important for developing feasible and sustainable community-based interventions that strengthen caregivers’ capacity to prevent childhood drowning.

To address these gaps, the Be SAFE Program was developed as a theory-based community learning intervention grounded in the HBM. The program consists of five structured learning modules integrating interactive health education, experiential learning activities, family participation, and continuous follow-up by VHVs. These components were designed to strengthen caregivers’ health beliefs, enhance self-efficacy, reduce perceived barriers, and reinforce cues to action, thereby supporting the sustained adoption of drowning prevention behaviors. By integrating behavioral theory with community participation and family-centered learning, the Be SAFE Program aims to address both the psychosocial determinants of preventive behavior and the practical challenges of implementing safe childcare practices in everyday life.

Given the continuing burden of drowning among young children and the central role of caregivers in preventing drowning incidents, effective community-based interventions that address both behavioral determinants and preventive practices are needed. Therefore, this study evaluated the effectiveness of the theory-based Be SAFE Program in improving HBM constructs and drowning prevention behaviors among caregivers of children younger than five years in community settings using a quasi-experimental design. We hypothesized that caregivers who participated in the Be SAFE Program would demonstrate significantly greater improvements in HBM constructs and drowning prevention behaviors than caregivers who received routine care and standard health education.

## 2. Materials and Methods

### 2.1. Study Design

This study employed a quasi-experimental pretest–posttest control group design. Participants were allocated to either an intervention group or a control group. The intervention group participated in the Be SAFE Program, whereas the control group received routine care and standard health education provided by Sub-district Health Promoting Hospitals (SHPHs) or relevant local agencies in the study area.

This study was conducted in Ubon Ratchathani Province, Thailand, which was purposively selected due to its high burden of childhood drowning. Subsequently, Phibun Mangsahan District was purposively selected as the study district because it reported the highest number of drowning deaths among children younger than five years in the province during 2019–2023.

Within the selected district, two SHPHs were selected from the available 18 SHPHs using a simple random sampling method with a non-replacement lottery procedure. The selected SHPHs were then randomly assigned to either the intervention group or the control group. This cluster-level allocation was applied because the intervention was delivered through SHPH healthcare teams and VHVs, thereby reducing the risk of contamination between participants receiving services within the same healthcare setting.

Following group allocation, eligible caregivers of children younger than five years were recruited from each participating SHPH according to the predefined inclusion and exclusion criteria.

### 2.2. Population and Sample

The accessible population comprised caregivers of children younger than five years who were registered within the catchment areas of two selected SHPHs in Phibun Mangsahan District, Ubon Ratchathani Province, Thailand. The final study sample included 60 caregivers, with 30 participants allocated to the intervention group and 30 to the control group.

Participants were selected using a multistage sampling technique. First, Phibun Mangsahan District was purposively selected because provincial injury surveillance data identified it as having the highest number of drowning deaths among children younger than five years during 2019–2023. Second, two SHPHs were randomly selected from the 18 SHPHs in the district using a simple random sampling method without replacement. The selected SHPHs were then randomly assigned to either the intervention group or the control group.

**Figure 1:**
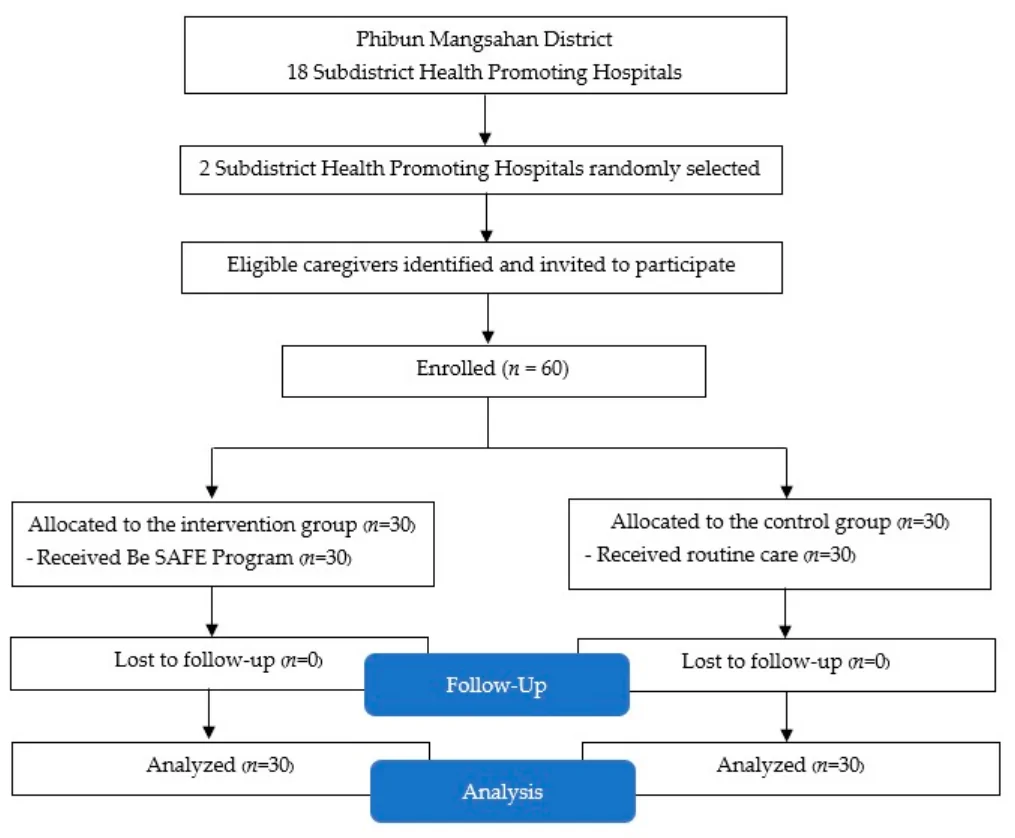
Flow diagram of participant recruitment, allocation, follow-up, and analysis.

Following the selection of the two SHPHs, eligible caregivers were identified with assistance from healthcare personnel and VHVs using local child health registration records. Caregivers who met the eligibility criteria were invited to participate voluntarily until the required sample size of 30 participants per group was achieved. The participant recruitment process is presented in Figure 1.

Participants were eligible if they (1) were parents or primary caregivers of children younger than five years, (2) were aged 18 years or older, (3) were able to read and write Thai, (4) had no physical disabilities or diagnosed mental health conditions that could interfere with participation, and (5) resided within the service areas of the selected SHPHs. Written informed consent was obtained from all participants before enrolment.

The required sample size was calculated based on the comparison of two independent means using data from a previous quasi-experimental study evaluating an HBM-based nurse-led drowning prevention program among caregivers in Ubon Ratchathani Province [28]. The effect size was derived from the mean differences reported in that study rather than being assumed a priori. Using G*Power version 3.1.9.7, with a significance level of 0.05, statistical power of 0.80, and an effect size of 0.865, the minimum required sample size was estimated to be 22 participants per group. To allow for potential attrition in the community setting, the sample size was increased by 35%, resulting in a target sample of 30 participants per group. Accordingly, 60 caregivers were enrolled, completed the intervention, and were included in the final analysis (Figure 1).

### 2.3. Instrument

The study instruments were developed based on a comprehensive review of the relevant literature and the HBM, where applicable. Because no standardized instrument was available to comprehensively assess the HBM constructs and drowning prevention behaviors targeted by the Be SAFE Program, a study-specific questionnaire was developed.

The questionnaire consisted of three sections: (1) sociodemographic characteristics; (2) perceptions based on the HBM constructs, including perceived susceptibility, perceived severity, perceived benefits, perceived barriers, cues to action, and self-efficacy; and (3) caregivers’ drowning prevention behaviors.

Content validity was evaluated by a panel of three experts with expertise in health education, child injury prevention, and behavioral science. Items with an Item–Objective Congruence (IOC) index of 0.50 or higher were retained, and the IOC values ranged from 0.67 to 1.00, indicating acceptable content validity.

Prior to the main study, the questionnaire was pilot-tested among 30 caregivers of children younger than five years from another district in Ubon Ratchathani Province who had characteristics similar to those of the target population. Internal consistency reliability was assessed using Cronbach’s alpha coefficients. The Cronbach’s alpha coefficients for the individual subscales ranged from 0.77 to 0.97, while the overall questionnaire demonstrated a Cronbach’s alpha coefficient of 0.79, indicating acceptable internal consistency reliability. The characteristics of each construct are presented in Table 1.

### 2.4. Intervention

The Be SAFE Program was developed as a theory-based, community-based learning intervention grounded in the HBM. The program title, “Be SAFE,” represents five structured learning modules: Better Life, Smart Protect, Able to Do, Family Action, and Encourage Behavior. These modules were designed to enhance caregivers’ safety awareness, family participation, empowerment, self-efficacy, and adoption of drowning prevention behaviors within the home environment.

The program was reviewed by a panel of five experts in public health, health education, and child injury prevention to evaluate its accuracy, utility, appropriateness, feasibility, and applicability. The overall program quality score was 4.73, indicating a very good level of quality. Additionally, the program manual and learning modules received mean quality scores of 4.80 and 4.93, respectively, also indicating very good quality.

The intervention consisted of five sequential learning modules (Table 2) delivered using interactive, participatory, and experiential learning approaches. Educational strategies included interactive lectures, multimedia presentations, group discussions, experience sharing, goal setting, persuasive communication, and home visits conducted by VHVs. Each module was designed to target specific HBM constructs through complementary learning activities. The sequence of the five learning modules was intentionally aligned with the HBM to progressively strengthen caregivers’ health beliefs, confidence, and motivation, thereby facilitating the adoption of effective drowning prevention behaviors.

The Be SAFE Program was implemented over an eight-week period. Modules 1–4 were delivered sequentially once per week during the first four weeks, with each session lasting approximately 90–120 min and facilitated by the principal investigator with support from trained VHVs. Module 5 consisted of follow-up home visits conducted by VHVs during weeks 6–8 to reinforce key messages, provide individualized guidance, monitor caregivers’ implementation of recommended practices, and support the application of recommended drowning prevention behaviors in the home environment. The post-intervention assessment was conducted after completion of the follow-up home visits at the end of week 8.

All participants in the intervention group attended all five Be SAFE educational sessions. Each participant received one follow-up home visit during weeks 6–8, and all visits were completed with the same participants who had completed the educational sessions. Attendance records and home-visit records were used to monitor participants’ exposure to the intervention. Although the intervention was delivered according to the predefined program schedule and intervention manual, a formal intervention fidelity assessment using standardized session checklists, independent observations, or other structured fidelity assessment procedures was not conducted, which is acknowledged as a limitation of the study.

### 2.5. Control Condition

Participants in the control group received routine care and standard health education provided by the SHPH. The health education covered the causes of drowning, potential consequences, and basic drowning prevention measures. It was delivered as a single face-to-face session during a routine home visit conducted as part of the SHPH’s standard public health services. Each session lasted approximately 20–30 min and used an interactive educational approach involving discussion and question-and-answer activities. The session was delivered by trained public health personnel at the SHPH.

### 2.6. Data Analysis

Data were analyzed using IBM SPSS Statistics for Windows, Version 27.0 (IBM Corp., Armonk, NY, USA). Descriptive statistics were used to summarize participants’ characteristics and study variables. Baseline sociodemographic characteristics between the intervention and control groups were compared using the chi-square test for categorical variables and the independent-samples *t*-test for continuous variables. Prior to analysis of covariance (ANCOVA), the assumptions of linearity, normality of residuals, homogeneity of variances, and homogeneity of regression slopes were assessed. The normality of continuous variables and model residuals was evaluated using the Kolmogorov–Smirnov test. Homogeneity of variances was assessed using Levene’s test, and homogeneity of regression slopes was examined by testing the interaction between the intervention group and the corresponding baseline score for each outcome variable. Post-intervention outcome scores were compared between the intervention and control groups using ANCOVA, with the post-intervention score as the dependent variable, study group as the fixed factor, and the corresponding baseline score as the covariate. Adjusted between-group mean differences with 95% confidence intervals (CIs), *p* values, and partial eta squared (η^2^p) were reported for each outcome. Because seven outcomes were analyzed, the Holm–Bonferroni sequential procedure was applied to control the family-wise error rate. Both unadjusted and Holm-adjusted *p* values are presented. Statistical significance was defined as a two-sided *p* value < 0.05.

## 3. Results

Most participants in both groups were female, married, had completed primary education, and cared for one child. In the intervention group, the mean age was 48.83 years (SD = 16.02). Most participants were married (73.4%), and more than half had completed primary education (60.0%). The mean monthly family income was 7913.33 Baht (SD = 3638.75). More than half of the participants cared for one child (53.4%), and grandparents constituted the predominant caregiver group. Similarly, participants in the control group were predominantly female, with a mean age of 46.43 years (SD = 13.60). Most participants were married (80.0%) and had completed primary education (53.4%). The mean monthly family income was 7900.00 Baht (SD = 3638.75). The majority cared for one child (76.7%), and grandparents were also the primary caregivers in this group. No statistically significant differences in sociodemographic characteristics were found between the two groups (Table 3).

All assumptions required for ANCOVA were assessed and found to be satisfied. The interaction between the study group and the corresponding baseline score was not statistically significant for any outcome variable (all interaction *p* > 0.05), indicating that the assumption of homogeneity of regression slopes was met. Furthermore, the Kolmogorov–Smirnov test indicated that the residuals were approximately normally distributed, and Levene’s test showed no evidence of heterogeneity of variances across groups for any outcome variable (all *p* > 0.05). Therefore, ANCOVA was considered appropriate for comparing post-intervention outcomes between the intervention and control groups after adjusting for the corresponding baseline scores. The baseline-adjusted comparisons are presented in Table 4.

Table 4 presents the baseline-adjusted comparisons of post-intervention outcomes between the intervention and control groups using ANCOVA, with the corresponding baseline scores included as covariates. After adjustment for baseline scores, caregivers in the intervention group had significantly higher adjusted mean scores for perceived susceptibility, perceived severity, perceived benefits, cues to action, self-efficacy, and drowning prevention behaviors than those in the control group (all Holm-adjusted *p* < 0.001). Conversely, the intervention group had significantly lower adjusted mean scores for perceived barriers than the control group (adjusted mean difference = −10.12, 95% CI: −11.10 to −9.14; Holm-adjusted *p* < 0.001). Adjusted mean differences ranged from 6.93 to 16.55 for outcomes with higher scores indicating improvement, whereas perceived barriers showed a reduction of 10.12 points. Effect sizes were consistently large across all outcomes (partial η^2^p = 0.785–0.964), indicating substantial short-term between-group differences after adjustment for baseline scores.

## 4. Discussion

The present study demonstrated that after adjustment for baseline scores using ANCOVA, caregivers who participated in the Be SAFE Program showed significantly greater short-term improvements in self-reported HBM constructs and drowning prevention behaviors than caregivers who received routine care. These between-group differences remained statistically significant after Holm adjustment for multiple comparisons and were accompanied by large effect sizes across all measured outcomes. Collectively, these findings suggest that the Be SAFE Program was associated with greater short-term improvements in caregivers’ health beliefs and self-reported drowning prevention behaviors than routine care.

The observed findings are consistent with the theoretical framework of the HBM, which proposes that individuals’ health beliefs are associated with the adoption of preventive health behaviors. The Be SAFE Program was specifically designed to address key HBM constructs through interactive educational activities, including multimedia presentations, group discussions, symbolic demonstrations, goal setting, persuasive communication, and follow-up home visits. Caregivers in the intervention group demonstrated higher post-intervention scores for perceived susceptibility, perceived severity, perceived benefits, cues to action, and self-efficacy, together with lower perceived barriers, compared with those receiving routine care. These findings are consistent with previous studies reporting that HBM-based educational interventions can improve caregivers’ health beliefs and child injury prevention behaviors [23,25,27,28,29,30].

The intervention incorporated multiple educational strategies that encouraged active participation and practical application of drowning prevention knowledge within the home environment. Interactive discussions, experience sharing, and contextualized learning activities may have helped caregivers better recognize drowning risks, understand the importance of preventive measures, and identify practical approaches for improving child supervision and home safety. Similarly, follow-up home visits conducted by VHVs provided opportunities to reinforce key educational messages and offer individualized guidance within participants’ home environments. However, because the present study did not evaluate the independent contribution of individual intervention components, the relative effectiveness of specific educational strategies or home visits cannot be determined.

Although caregivers in the intervention group demonstrated substantially higher self-reported self-efficacy scores than those in the control group following the intervention, the present findings should not be interpreted as evidence that improvements in self-efficacy caused the observed behavioral improvements. Likewise, although the intervention was developed according to the HBM and improvements were observed across multiple HBM constructs and drowning prevention behaviors, the present study did not assess the temporal sequence of these changes or conduct mediation analyses. Therefore, the observed improvements should be interpreted as concurrent short-term changes rather than evidence that changes in HBM constructs directly mediated behavioral change.

The present findings are consistent with previous studies demonstrating that theory-based educational interventions can improve caregivers’ perceptions and child injury prevention practices [23,25,27,28,29,30]. They also support existing evidence emphasizing the importance of caregiver supervision, home environmental safety, and community-based education in reducing childhood drowning risks [21,31,32]. However, because drowning events, injuries, and mortality were not measured, the potential contribution of the Be SAFE Program to reducing drowning-related outcomes remains hypothetical and should be evaluated in future research.

Although modest improvements may occur through routine care and standard health education, the baseline-adjusted analyses demonstrated significantly greater short-term improvements across all measured HBM constructs and drowning prevention behaviors among caregivers participating in the Be SAFE Program. These findings suggest that a structured, theory-informed educational program may provide additional benefits beyond routine health education. Nevertheless, the present study was not designed to determine which specific intervention components were responsible for the observed improvements.

The findings have several practical implications for community health promotion. The Be SAFE Program demonstrates the feasibility of delivering a structured HBM-based educational intervention through existing primary healthcare services and community health networks, including VHVs. This approach aligns with evidence highlighting that sustainable drowning prevention requires coordinated actions across healthcare systems, communities, and other sectors [32]. These findings suggest that such programs may have potential for integration into maternal and child health services in similar community settings. However, further evidence from larger studies involving multiple sites, randomized or cluster-randomized designs, and longer follow-up periods is required before broader implementation can be recommended.

This study has several strengths. The intervention was developed using a well-established behavioral theory and culturally appropriate learning activities designed for caregivers of children younger than five years. The program was implemented through existing community health structures, including VHVs, thereby enhancing the practicality and relevance of the intervention within the local primary healthcare context. In addition, the use of baseline-adjusted ANCOVA with Holm correction strengthened the statistical comparison between study groups by accounting for baseline differences and controlling the family-wise error rate across multiple outcomes.

Several limitations should be acknowledged. First, the study employed a quasi-experimental design in which participants in the intervention and control groups were recruited from two SHPHs per study arm. Although baseline-adjusted ANCOVA was performed, the observed between-group differences cannot be fully separated from potential site-level differences. Therefore, the findings should be interpreted as exploratory. Second, the study was conducted in a single district of Ubon Ratchathani Province using purposive sampling, which may limit the generalizability of the findings to other settings. Third, all outcomes were assessed using self-reported questionnaires and may therefore be subject to reporting bias. Fourth, although participant attendance and home-visit completion were monitored, no formal assessment of intervention fidelity was conducted using standardized fidelity measures. Finally, the relatively short follow-up period precluded evaluation of the longer-term maintenance of the observed improvements.

Future studies should include larger samples from multiple provinces, a greater number of participating SHPHs, and randomized or cluster-randomized designs with longer follow-up periods. In addition, future research should incorporate objective measures of drowning prevention practices and drowning-related outcomes, evaluate intervention fidelity using standardized procedures, and conduct mediation analyses to better understand the relationships between HBM constructs and behavioral outcomes.

Overall, the present findings suggest that the Be SAFE Program was associated with greater short-term improvements in caregivers’ self-reported HBM constructs and drowning prevention behaviors than routine care after adjustment for baseline scores. These findings support the potential value of HBM-based community education for childhood drowning prevention while highlighting the need for larger, multi-site studies with longer follow-up to confirm the effectiveness and broader applicability of the program.

## 5. Conclusions

The findings of this study suggest that the HBM-based Be SAFE Program was associated with greater short-term improvements in caregivers’ self-reported health beliefs and drowning prevention behaviors than routine care after adjustment for baseline scores. Caregivers who participated in the program demonstrated significantly greater improvements across multiple HBM constructs, including perceived susceptibility, perceived severity, perceived benefits, cues to action, and self-efficacy, together with lower perceived barriers and improved self-reported drowning prevention behaviors. These findings suggest that structured community-based educational programs grounded in the HBM may have potential for strengthening childhood drowning prevention within primary healthcare settings.

The Be SAFE Program represents a feasible community-based educational approach that may be integrated into routine maternal and child health services in similar settings. However, because this study used a quasi-experimental design with a relatively small sample and short follow-up period, larger multi-site studies using randomized or cluster-randomized designs, longer follow-up, objective outcome measures, and mediation analyses are needed to confirm the program’s effectiveness, evaluate its long-term sustainability, and determine whether improvements in caregiver behaviors are associated with reductions in drowning-related injuries and mortality.

## Figures and Tables

**Table 1:** Structure, scoring, and reliability of the study questionnaire.

Section/ Construct	Example of Items	Response Scale	Number of Items	Range of Scores	Cronbach’s Alpha
Section 2 Health Belief Model constructs		5-point Likert scale (1 = Strongly disagree to 5 = Strongly agree)	40	-	-
Perceived susceptibility	Young children are at risk of drowning even in containers such as buckets or basins containing only 1–2 inches (approximately 2.5–5 cm) of water.		6	6–30	0.88
Perceived severity	A child who is not rescued within four minutes after drowning may suffer severe brain damage or remain in a permanent vegetative state.		6	6–30	0.85
Perceived benefits	Emptying water containers after use and keeping water storage containers covered can help prevent drowning among young children.		6	6–30	0.86
Perceived barriers	I feel that supervising my child without engaging in other activities (e.g., using a mobile phone or doing household chores) is time-consuming.		6	6–30	0.97
Cues to action	I empty water containers after use and keep water storage containers covered every time, following the recommendations of Village Health Volunteers (VHVs).		6	6–30	0.92
Self-efficacy	I am confident that I can closely supervise my child and never leave him or her alone in or near water.		10	10–50	0.96
Section 3 Drowning prevention behaviors		4-point Likert scale (1 = Never to 4 = Every day/Every time)			
Drowning prevention behaviors	I supervise my child by staying within sight and close enough to reach him or her immediately if necessary.		6	6–24	0.77

**Table 2:** Overview of the Be SAFE program and corresponding HBM constructs.

Modules	Activity Title	Content	Activities	Targeted HBM Constructs
1	Better Life	Childhood drowning situation among children younger than 5 years Causes and risk factors of childhood drowning Consequences and severity of drowning	Interactive lecture with PowerPoint presentation Symbolic model demonstration Group discussion on drowning risks	Enhancing perceived susceptibility and perceived severity
2	Smart Protect	Importance of childhood drowning prevention Residential drowning prevention measures Common barriers to implementing preventive behaviors	Interactive lecture Short educational video Small-group brainstorming Group discussion	Strengthening perceived benefits and reducing perceived barriers
3	Able to Do	Close supervision of children younger than 5 years Environmental safety management around the home	Interactive lecture Demonstration using symbolic models Group discussion Persuasive communication and experience sharing	Enhancing self-efficacy
4	Family Action	Reliable sources of drowning prevention information Practical recommendations for preventing childhood drowning	Reflection and experience sharing Information provision Goal setting and personal commitment	Promoting cues to action
5	Encourage Behavior	Continuous child supervision practices Safe home environmental management Accessing drowning prevention information resources	Home visits Motivational discussion Verbal encouragement and reinforcement	Supporting drowning prevention behaviors through follow-up support and behavioral reinforcement

**Table 3:** Baseline sociodemographic characteristics of participants in the intervention and control groups.

Sociodemographic Characteristics	Intervention Group (*n* = 30)		Control Group (*n* = 30)		Statistical Analysis
	*n*	%	*n*	%	
Sex					
Female	24	80.0	23	76.7	χ^2^ = 0.098 *p* = 0.754
Male	6	20.0	7	23.3	
Age mean ± SD (y)	48.83 ± 16.020		46.43 ± 13.602		t = 0.626 *p* = 0.534
Marital Status					
Single	4	13.3	3	10.0	χ^2^ = 0.479 *p* = 0.829
Married (Couple)	22	73.4	24	80.0	
Widowed/Divorced/Separated	4	13.3	3	10.0	
Education Level					
Primary School	18	60.0	16	53.4	χ^2^ = 3.477 *p* = 0.445
Lower Secondary School	8	26.7	5	16.7	
Upper Secondary School	4	13.3	7	23.3	
Associate Degree or Equivalent	0	0.0	1	3.3	
Bachelor’s Degree or Higher	0	0.0	1	3.3	
Monthly Family Income mean ± SD (baht)	7913.33 ± 3638.751		7900.00 ± 3693.891		t = 0.014 *p* = 0.989
Number of Children Under Care (All children under care)					
1 child	16	53.4	23	76.7	χ^2^ = 5.474 *p* = 0.057
2 children	10	33.3	7	23.3	
3 children	4	13.3	0	0.0	
Relationship to Child Under Care					
Mother	9	30.0	11	36.6	χ^2^ = 0.880 *p* = 0.699
Grandparent	17	56.7	17	56.7	
Aunt/Uncle	4	13.3	2	6.7	

**Table 4:** Baseline-adjusted comparisons of post-intervention outcomes between the intervention and control groups using ANCOVA.

Outcome	Adjusted Mean (SE) Intervention	Adjusted Mean (SE) Control	Adjusted Mean Difference (95% CI)	ANCOVA *F* (df1, df2)	*p*	Holm-Adjusted *p*	Partial η^2^p
Perceived susceptibility	27.23 (0.33)	20.30 (0.33)	6.93 (5.97, 7.89)	F(1, 57) = 208.08	<0.001	<0.001	0.785
Perceived severity	27.56 (0.25)	19.59 (0.25)	7.96 (7.24, 8.68)	F(1, 57) = 491.53	<0.001	<0.001	0.896
Perceived benefits	27.68 (0.26)	18.54 (0.26)	9.14 (8.39, 9.89)	F(1, 57) = 597.09	<0.001	<0.001	0.913
Perceived barriers	8.52 (0.34)	18.64 (0.34)	−10.12 (−11.10, −9.14)	F(1, 57) = 429.37	<0.001	<0.001	0.883
Cues to action	27.08 (0.30)	19.04 (0.30)	8.03 (7.17, 8.89)	F(1, 57) = 347.31	<0.001	<0.001	0.859
Self-efficacy	47.47 (0.29)	30.92 (0.29)	16.55 (15.70, 17.39)	F(1, 57) = 1538.94	<0.001	<0.001	0.964
Drowning prevention behaviors	22.05 (0.23)	14.17 (0.23)	7.87 (7.20, 8.55)	F(1, 57) = 548.36	<0.001	<0.001	0.906

Abbreviations: ANCOVA = analysis of covariance; SE = standard error; CI = confidence interval; η^2^p = partial eta squared. Adjusted means were estimated from ANCOVA models with study group as the fixed factor and the corresponding baseline score as the covariate. Adjusted mean differences represent baseline-adjusted differences between the intervention and control groups. Holm-adjusted *p* values were calculated using the Holm–Bonferroni sequential procedure to control the family-wise error rate across the seven outcome variables.

## Data Availability

The data presented in this study are available from the corresponding author upon reasonable request. The data are not publicly available due to privacy and ethical restrictions related to human participants.

## Abbreviations

The following abbreviations are used in this manuscript:

HBM	Health Belief Model
SHPH	Subdistrict Health Promoting Hospital
VHV	Village Health Volunteer
ANCOVA	Analysis of Covariance
